# Using Secure Multi-Party Computation to Protect Privacy on a Permissioned Blockchain

**DOI:** 10.3390/s21041540

**Published:** 2021-02-23

**Authors:** Jiapeng Zhou, Yuxiang Feng, Zhenyu Wang, Danyi Guo

**Affiliations:** School of Software Engineering, South China University of Technology, Guangzhou 510006, China; 201821038647@mail.scut.edu.cn (J.Z.); wangzy@scut.edu.cn (Z.W.); 201821038605@mail.scut.edu.cn (D.G.)

**Keywords:** privacy, secure multi-party computation, permissioned blockchain, Hyperledger Fabric

## Abstract

The development of information technology has brought great convenience to our lives, but at the same time, the unfairness and privacy issues brought about by traditional centralized systems cannot be ignored. Blockchain is a peer-to-peer and decentralized ledger technology that has the characteristics of transparency, consistency, traceability and fairness, but it reveals private information in some scenarios. Secure multi-party computation (MPC) guarantees enhanced privacy and correctness, so many researchers have been trying to combine secure MPC with blockchain to deal with privacy and trust issues. In this paper, we used homomorphic encryption, secret sharing and zero-knowledge proofs to construct a publicly verifiable secure MPC protocol consisting of two parts—an on-chain computation phase and an off-chain preprocessing phase—and we integrated the protocol as part of the chaincode in Hyperledger Fabric to protect the privacy of transaction data. Experiments showed that our solution performed well on a permissioned blockchain. Most of the time taken to complete the protocol was spent on communication, so the performance has a great deal of room to grow.

## 1. Introduction

Traditional centralized systems provide efficient and personalized service, but the negative effects of centralization are increasingly appearing: corruption, inequality and privacy issues. As it turns out, some decentralized technologies [1] are urgent. Blockchain is a peer-to-peer and decentralized ledger technology that has the characteristics of transparency, consistency, immutability and traceability. First popularized for crypto-currency systems such as Bitcoin [2], blockchain has seen explosive development in recent years. There are two types of blockchain: public and permissioned. Anyone can freely join a public blockchain and submit proposals, whereas a permissioned blockchain is dominated by a group of known nodes and restricts joining the network via access control.

A core problem is that all users who have joined the blockchain see an identical ledger, making it thorny to handle transactions that rely on confidential data [3]. Access control mechanisms are usually used to deal with the privacy requirements of the associated stakeholders in decentralized networks [4] such as blockchains. One instance is the Hyperledger Fabric Channel, which protects privacy by restricting data access, but the problem still exists, resulting from the fact that nodes in the same channel deal with identical transactions. A simpler solution is public key cryptography. In this solution, the participant encrypts a message using his public key and submits it to the ledger, but ciphertexts under different public keys can not be collaboratively analyzed. The privacy issues limit the wide application of blockchain.

Excitingly, the cryptographic technology of secure multi-party computation is a perfect way to deal with the problem of privacy. This concept dates back to what is called “Yao’s millionaires’ problem” [5], a famous problem introduced by Andrew Yao in 1982. Formally, we assume that n participants ($P_{1},P_{2},\ldots ,P_{n}$) all hold the secret data $x_{1},x_{2},\ldots ,x_{n}$ and that they are willing to cooperate to compute a function $\left(y_{1},y_{2},\ldots ,y_{n}\right)$ ← $F(x_{1},x_{2},\ldots ,x_{n})$. Throughout the whole process, each participant $P_{i}$ only learns his own value $x_{i},y_{i}$, and information can be derived from $y_{i}$. Beyond that, he learns nothing. Many studies have been undertaken in order to design secure multi-party computation (MPC) protocols, such as oblivious transfer [6], garbled circuit [7], homomorphic encryption [8] and the linear secret sharing scheme [9,10].

Secure MPC provides enhanced privacy, correctness and independence of inputs, and guarantees output delivery. Blockchain perfectly suits secure MPC protocols because they all deal with security and trust issues in distributed environments [11]. There are many practical scenarios that benefit from utilizing secure MPC based on blockchain, such as statistical analysis of health data [12], anonymous electronic voting [13], initial public offering(IPO) [3] and edge computing [14].

In this paper, we propose a publicly verifiable, secure MPC protocol to protect the transaction privacy of a permissioned blockchain. The protocol contains two parts: an on-chain computation phase and an off-chain preprocessing phase. Operations such as key generation, data encryption and generating pre-processing data are implemented off-chain, and secure computations are performed on-chain. The execution of the on-chain protocol is integrated as part of the chaincode in Hyperledger Fabric [15]. Concretely, this paper makes the following contributions:

(1) In the on-chain phase, we facilitate additive secret sharing and the Paillier cryptosystem, an additive homomorphism encryption scheme, to preserve the confidentiality of private data. Computations are based on encrypted shares and can be parallelized, which greatly resolves the N-1 attack problem. We integrated the on-chain protocol as part of the smart contract to utilize its correctness and verifiability. (2) We adopted zero-knowledge proof technology, specifically that of Pedersen [16], to construct a non-interactive verifiable secret sharing scheme and to prove the correctness of calculation tasks. Any stage and any intermediate result can be verified by opening commitments. (3) In the off-chain phase, based on Beaver randomization technology [17], we designed a new protocol to generate <a,b,c,d> quadruples, making it possible to perform multiplication on encrypted secret shares (differently from SPDZ [18]). (4) Finally, we describe how to integrate our secure MPC protocol into Hyperledger Fabric. By adding a pluggable component called "decryptor" to Hyperledger Fabric, we allow peers to process decryption requests during the endorsement phase, which eliminates communication interactions among participants and the blockchain. For as long as decryptors are online, what participants need to do is encrypt, input and wait for outputs.

The remainder of the paper is structured as follows. In Section 2, previous studies on privacy protection in blockchains are briefly reviewed. In Section 3, details of the proposed method are described. Section 4 discusses the security issues surrounding the proposed method. In Section 5, experimental results of the computational performance are presented. In Section 6, we provide our conclusions on the work presented in this paper.

## 2. Related Work

Many works have been done to protect privacy in the blockchain in recent years. Frequently used cryptographic techniques [19] for privacy protection in blockchains [20,21,22,23] include ring signature, mixing services and zero-knowledge proof.

Here we mainly focus on investigating the development of the combination of blockchain and secure MPC. Bitcoin was first utilized to obtain fairness in a secure multi-party protocol [24,25,26]. Other researchers have also endeavored to deploy secure MPC on blockchains to solve the problem of privacy.

Sánchez [27] considered outsourcing encrypted computation to cloud-based blockchain and rewarding miners for generating preprocessing data for secure multi-party computation. To provide correctness, verifiability and privacy confidentiality for smart contracts in the blockchain, they combined proof-carrying code and secure multi-party computation, which effectively handle Gyges and DAO attacks. In addition, zero-knowledge proofs of proofs is used to prove the validity of smart contracts. Enigma, proposed by Zyskind et al. [28], is a blockchain-based decentralized computation platform. Their architecture consists of two parts: blockchain and Enigma. Enigma is an off-chain network responsible for private and intensive computations. Blockchain deals with access control, identity management, link protocols and the tamper-proof log of events in Enigma. They made a series of performance improvements to secure MPC, such as hierarchical secure MPC, adaptable circuits and network reduction, making the technology practical even when used in a large network. Kosba et al. [29] proposed HAWK, a user-friendly framework for creating smart contracts with guaranteed privacy. Hawk provides a compiler with which programmers have no necessity to implement any cryptography. It relies on a trusted manager to handle confidential data and the manager is trusted not to leak secrets, which can be implemented by trusted hardware or instantiated with multi-party computation. Choudhuri et al. [30] used witness encryption to transform the fairness problem in secure MPC into the one in decryption. They utilized bulletin boards such as Google’s certificate transparency logs and a blockchain to record some publicly untamable information. Participants need to release to the bulletin board tokens with shares, which others could use to decrypt ciphertext, and then they must publish their secret in limited time.

Some researchers focused on Hyperledger Fabric, a typical permissioned blockchain hosted by the Linux Foundation. Benhamouda et al. [31] used secure MPC to support private-data computation in Hyperledger Fabric. In contrast to previous studies, they integrated secure MPC protocols as part of the smart contract rather than running them in an off-chain network. However, their solution requires “privileged clients” that have access to the same private key peers used for data encryption. Ghadamyari et al. [12] also focused on Hyperledger Fabric. Although they facilitated the Paillier cryptosystem to obtain data privacy and used access control list rules to restrict access to the ledger, data owned by different participants are encrypted by the same public key, resulting in disclosing privacy when the owner of the private key and the invoker of the smart contract conspire.

The previous work can be classified into two main types: on-chain secure MPC protocols that integrate the protocols into the blockchain architecture itself, and off-chain secure MPC protocols that offload intensive computation to an off-chain network. Benhamouda [31] described a comparison between these two types of protocols, and we briefly sum that up as follows: Running the secure MPC on-chain enables us to take use of the blockchain facilities for communication and identity management, which need to be re-implemented in the off-chain secure MPC protocol. The core advantage of an off-chain secure MPC protocol is its efficiency of computation, but the situation is more applicable to permissionless blockchains, which are typically slower than permissioned ones. Thus, on-chain secure MPC protocols are more applicable to be deployed on permissioned blockchains, such as Hyperledger Fabric.

Our MPC-over-Fabric architecture is similar to the on-chain secure MPC protocol described by Benhamouda [31], but not quite the same. One key difference is that we do not require “privileged clients” or a “helper server”, which may raise some security concerns. Without any security assumptions, we add a pluggable component called a “decryptor” to the peer responsible for decrypting during the endorsement phase. Another difference is that secrets belong to the participants—the data providers who take part in computation through clients of the blockchain. Participants split their secrets into shares and encrypt these shares using different public keys, ensuring no single participant can see all of them. Endorsement peers only execute smart contracts, but those in [31] also serve as the entities with the secrets.

## 3. The Proposed Method

### 3.1. Overview of Our Framework

**Figure 1:**
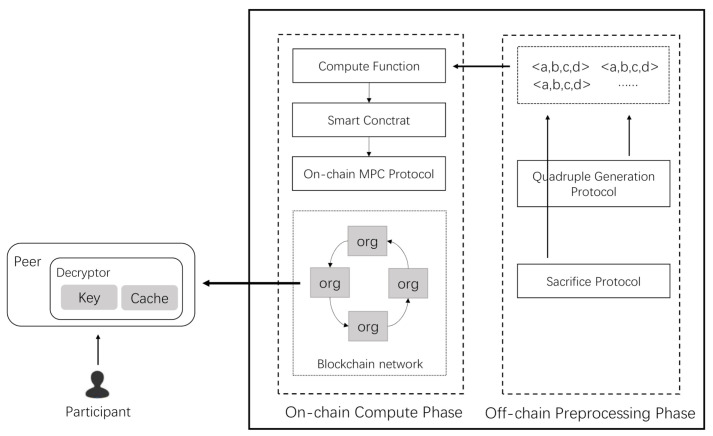
Architecture overview of the secure MPC scheme. Participants interact with the blockchain through peers, which have joined in the organizations in the blockchain.

In this section, we describe the main framework of the proposed secure MPC computation scheme. Figure 1 shows the proposed architecture for secure MPC based on a blockchain, which contains two phases: an on-chain computation phase and an off-chain preprocessing phase. Operations such as generating preprocessing data, key generation and data encryption are implemented off-chain, and secure computations are performed on-chain. The execution of MPC computation protocol is integrated as part of the chaincode in Hyperledger Fabric.

The related parties are briefly explained as follows:

- **Participant**: the data owner.
- **Peer**: compute node in Hyperledger Fabric, concretely the endorsement node.
- **Decryptor**: a component in the Peer assisting decrypting during the endorsement phase. (details can be seen in Section 3.4.3).
- **Compute function**: the computational logic provided by participants.
- **On-chain MPC protocol**: the auxiliary protocol utilized to perform on-chain, secure, multi-party computation.
- **Quadruple generation protocol**: the protocol used to generate preprocessing data (<a,b,c,d> quadruples) for on-chain secure multi-party computation.
- **Sacrifice protocol**: the protocol used to check whether a quadruple is valid.

A jointly secure computation task proceeds according to the following steps:

1. (Off-chain preprocessing phase): All participants use the quadruple generation protocol to obtain a sufficient number of <a,b,c,d> quadruples and check whether the quadruples are valid using the sacrifice protocol. This step is not necessary for every task. A huge number of quadruples can be generated in advance for future tasks.
2. (On-chain computation phase): All participants break raw input values to secret shares, encrypt secret shares and quadruple shares, generate commitments and submit all of them to the ledger.
3. (On-chain computation phase): Participants store the addresses of decryptors to the ledger.
4. (On-chain computation phase): The invoker (one of the participants) invokes the MPC smart contract to execute the computation task.
5. (On-chain computation phase): Participants decrypt their outputs, which can also be carried out by decryptors, and reconstruct the final result.

### 3.2. The Algorithms Used

In this section, we describe the cryptography algorithms used in our protocol.

(1) Additive homomorphism encryption

Additive homomorphic encryption allows one to perform computations on ciphertext. The result of the calculation is the encryption of their sum.

Let AHE = (KeyGen, Enc, Dec, Add, Sub, cMul) be an instance of an asymmetric additive homomorphism encryption scheme. We will use the following definitions: The message space is denoted as $\tilde{M}$, and the ciphertext space is denoted as $\tilde{C}$. ⊕ denotes the addition of $\tilde{C}$ and ⊖ denotes the subtraction of $\tilde{C}$. The multiplication of a scalar plaintext $m\in \tilde{M}$ and a ciphertext $c\in \tilde{C}$ is denoted as ⊗, and the encryption of the plaintext message m is denoted as $\bar{m}\in \tilde{C}$. AHE is denoted as follows:

1. KeyGen(κ): takes the security parameter κ as the input, and outputs the private key sk and the public key pk.
2. Enc(pk, m): takes the message $m\in \tilde{M}$ and the public key pk as the inputs, and outputs the ciphertext $\bar{m}\in \tilde{C}$.
3. Dec(sk, c): takes the ciphertext $c\in \tilde{C}$ and a private key sk as the inputs, and outputs the decrypted result $m\in \tilde{M}$.
4. Add(c1, c2): takes two ciphertexts $c1,c2\in \tilde{C}$ as the inputs, and outputs the ciphertext c3=c1⨁c2, so that Dec(sk,c3)=Dec(sk,c1)+Dec(sk,c2)
5. Sub(c1, c2): takes two ciphertexts $c1,c2\in \tilde{C}$ as the inputs, and outputs the ciphertext c3=c1⊖c2, so that Dec(sk,c3)=Dec(sk,c1)−Dec(sk,c2)
6. cMul(m1, c1): takes the scalar $m1\in \tilde{M}$ and the ciphertext $c1\in \tilde{C}$ as the inputs, and outputs the ciphertext $c2=m1⨂c1\in \tilde{C}$, so that Dec(sk,c2)=m1∗Dec(sk,c1)

In this paper, we use the popular Paillier [32] cryptosystem as the additive homomorphism encryption scheme due to its simple structure and high execution efficiency. It has been applied to many practical scenarios, such as electronic voting and federated learning. The Paillier cryptosystem has the plaintext space $Z_{N}$ and the ciphertext space $Z_{N^{2}}$*, and *N* is the security parameter.

(2) Additive secret sharing

In this paper, we use (*n*,*n*)-threshold additive secret sharing scheme and its implementation is as follows [33].

1. Secret shares: To share a secret a, the dealer chooses n−1 random shares $a_{j}$ (j=1,2,…,n−1) in GF(*p*), and computes $a=a_{n}+\sum_{j=1}^{n-1}a_{i}\;\left(mod\;p\right)$. The dealer then sends the shares $a_{i}$ to participant $P_{i}$ (i=1,…,n).
2. Secret reconstruction: All participants collaboratively reconstruct the secret: $a=\sum_{j=1}^{n}a_{i}\;\left(mod\;p\right)$.

The above implementation possesses additive homomorphism. Concretely, if participants $P_{1},P_{2},\ldots ,P_{n}$, respectively, hold shares $a_{1},a_{2},\ldots ,a_{n}$ and $b_{1},b_{2},\ldots ,b_{n}$ that are associated with secrets *a* and *b*, the shares of (a+b) are $a_{1}+b_{1},a_{2}+b_{2},\ldots ,a_{n}+b_{n}$.

(3) Pedersen

Pedersen [16,34] is a non-interactive commitment scheme with additive homomorphism, so we can easily perform the same calculation on commitments to prove the correctness of the results at any stage. We use Pedersen to construct our non-interactive verifiable secret sharing scheme. There are three phases in the Pedersen scheme used in our proposed method:

1. Setup Phase: All participants agree on an elliptic curve *E* over a field $F_{n}$, a generator $G\in E/F_{n}$ and $H\in E/F_{n}$.
2. Commitment Phase: Participant $P_{i}$ chooses a random number $r\in F_{n}$, and computes the point $Comm_{i}=C\left(x_{i},r\right)=r*G+x_{i}*H$, which represents the commitment for $P_{i}$’s secret $x_{i}$. *r* is a blinding factor that prevents observers from guessing $x_{i}$. $P_{i}$ sends $Comm_{i}$ to the receiver $P_{j}$.
3. Open Phase: $P_{i}$ sends ($x_{i}$, r) to the receiver $P_{j}$ and $P_{j}$ verifies whether $r*G+x_{i}*H$ equals $Comm_{i}$. $P_{j}$ refuses the commitment if they are not equal.

The Pedersen algorithm also has additive homomorphism and it is easy to prove the following equations:

$$\begin{array}{c} Comm(v1+v2,r1+r2)=Comm(v1,r1)+Comm(v2,r2)\\ Comm(v1-v2,r1-r2)=Comm(v1,r1)-Comm(v2,r2)\\ Comm(c*v,c*r)=c*Comm(v,r) \end{array}$$

### 3.3. The Secure MPC Protocol

In this section, we present the core idea of our secure MPC protocol. Since a blockchain is completely public, we aim to build a protocol for multi-party arithmetic computation over $F_{p}^{k}$, which is composed of addition and multiplication, and has the following characteristics:

1. Privacy confidentiality: All the input values and output values are in encrypted form and no one learns anything except his own input secret and output values.
2. Publicly verifiable: Computations can be publicly executed and are controlled by no one. Any step during computation can be publicly verified.

Similarly to SPDZ [18], structured in an offline phase and an online phase, our solution consists of an on-chain computation phase and an off-chain preprocessing phase. We combine additive secret sharing and the Paillier cryptosystem to preserve the confidentiality of privacy. Computations are based on encrypted shares, which can be parallelized. What is more, the Pedersen system is utilized to ensure the verifiability of secret sharing and computations.

We assume a publicly trusted processor (*PTP*), which can be instantiated with a trusted execution environment (TEE) or a trusted player. In this paper, the smart contract in the blockchain plays the role of *PTP*. *PTP* receives inputs, calculates functions and generates outputs without mistakes. All behaviors of *PTP* are public and can be verified.

#### 3.3.1. On-Chain Computation Procotol

The on-chain computation phase consists of four sub-protocols—protocol input, protocol addition, protocol multiplication and protocol output. We assume enough <a,b,c,d> quadruples have been generated in the off-chain preprocessing phase. Details about quadruple generation can be seen in Section 3.3.2.

(1) 1. **Protocol Input**

1. All participants $P_{i}\;\left(i=1,2,\ldots ,m\right)$ use the Paillier algorithm to generate their private key $sk_{i}$ and public key $pk_{i}$.
2. Each participant $P_{i}$ serves as a dealer, who uses additive secret sharing to break his secret $S_{i}$ to n shares $S_{ij}$ (j=1,2,…,n), and then $P_{i}$ encrypts the share for $P_{j}$ using $P_{j}$’s public key:
  (1) $$\bar{S_{ij}}\leftarrow Enc\left(pk_{j},S_{ij}\right),j=1,2,\ldots ,n$$
3. $P_{i}$ chooses *n* random blinding factor $x_{ij}\in F_{n}$(j=0,1,2,…,n), and computes the commitments for shares $S_{ij}$(j=1,2,…,n):
  (2) $$Comm_{ij}=C\left(S_{ij},x_{ij}\right)\leftarrow x_{ij}*G+S_{ij}*H,j=1,2,\ldots ,n$$
4. $P_{i}$ encrypts the blinding factor:
  (3) $$\bar{x_{ij}}\leftarrow Enc\left(pk_{j},x_{ij}\right),j=1,2,\ldots ,n$$
5. $P_{i}$ generates the commitment of the secret value itself:
  (4) $$Comm_{i}=C\left(S_{i},x_{0}\right)\leftarrow x_{0}*G+S_{i}*H,\;x_{0}=\sum_{k=1}^{n}x_{k}$$
6. Finally, $P_{i}$ submits his input
  (5) $$\left\{\bar{S_{i1}},\bar{S_{i2}},\ldots ,\bar{S_{in}},Comm_{i},Comm_{i1},Comm_{i2},\ldots ,Comm_{in},\bar{x_{i1}},\bar{x_{i2}},\ldots ,\bar{x_{in}}\right\}$$
  to *PTP*.

Other participants $P_{j}\left(j\neq i\right)$ can freely get $P_{i}$’s input from *PTP* and decrypt $\bar{S_{ij}},\;\bar{x_{ij}}$ using his secret key $sk_{j}$, and then open and verify the commitments by checking whether the following equations are all true:

(6) $$\begin{array}{cc} Dec\left(sk_{j},\bar{x_{ij}}\right)*G & +\;Dec(sk_{j},\bar{S_{ij}})*H=\;Comm_{ij}\\ & Comm_{i}=\sum_{k=1}^{n}Comm_{ik} \end{array}$$

If true, the share $S_{ij}$ is valid and $P_{i}$ splits his secret value correctly, meaning the $P_{i}$ has honestly submitted his input.

Moreover, participants must submit enough quadruples <a,b,c,d> and commitments of quadruples shares to *PTP* before computation. $P_{i}$ encrypts his quadruple shares using his public key $pk_{i}$ and submits it to *PTP*:

(7) $$<\bar{a_{ik}},\bar{b_{ik}},\bar{c_{ik}},\bar{d_{ik}}>\leftarrow Enc\left(sk_{i},<a_{ik},b_{ik},c_{ik},d_{ik}>\right),\;k\;is\;the\;index\;of\;quadruples$$

(2) **Protocol Addition**

An addition gate is defined as follows:

(8) $$\begin{array}{c} \begin{array}{cc} \left\{\bar{y_{1}},\bar{y_{2}},\ldots ,\bar{y_{n}}\right\} & \leftarrow \tilde{F}\left(\bar{<S_{1}>},\bar{<S_{2}>},\ldots ,\bar{<S_{m}>}\right)\\ \bar{<S_{k}>} & \leftarrow (\bar{S_{k1}},\bar{S_{k2}},\ldots ,\bar{S_{kn}}),\;k=1,2,\ldots ,m \end{array} \end{array}$$

The inputs are the encrypted shares of secret inputs $S_{i}$ (i=1,2,…,m), and there are n output values for *n* participants. The *i*-th output value $\bar{y_{i}}$ is a ciphertext encrypted by $P_{i}$’s public key $pk_{i}$. We define the addition gate as computation of any linear function:

(9) $$F\left(\cdot \right)\leftarrow \sum_{i=1}^{m}c_{i}*S_{i}$$

1. To compute $S_{i}+S_{j}$, *PTP* computes:
  (10) $$\bar{y_{k}}=\bar{S_{ijk}}\leftarrow \bar{S_{ik}}⊕\bar{S_{jk}},\;k=1,2,\ldots ,n$$
2. For a public scalar c∈GF(p) and a secret input $S_{i}$, to compute $c*S_{i}$, *PTP* computes:
  (11) $$\bar{y_{k}}=\bar{S_{cik}}\leftarrow c⊗\bar{S_{ik}},k=1,2,\ldots ,n$$
3. To compute $c+S_{i}$, *PTP* computes:
  (12) $$\begin{array}{c} \bar{y_{k}}=\bar{S_{cik}}\leftarrow \bar{c_{k}}⊕\bar{S_{ik}}\;,\;k=1,2,\ldots ,n\\ (\bar{c_{1}}=Enc\left(sk_{1},c\right),\;\bar{c_{j}}=Enc\left(sk_{j},0\right)\;\left(j=2,\ldots ,n\right)) \end{array}$$

Since both (*n*,*n*)-threshold additive secret sharing and the Paillier cryptosystem are additively homomorphic, any linear function with *n* inputs can be computed locally without interactions.

Then, we can prove the equation:

(13) $$F\left(S_{1},S_{2},\ldots ,S_{m},c_{1},c_{2},\ldots ,c_{m}\right)=\sum_{k=1}^{n}Dec(sk_{k},\bar{y_{k}})$$

1. For $S_{i}+S_{j}$:
  (14) $$\sum_{k=1}^{n}Dec(sk_{k},\bar{y_{k}})=\sum_{k=1}^{n}\left(S_{ik}+S_{jk}\right)=S_{i}+S_{j}$$
2. For $c*S_{i}$:
  (15) $$\sum_{k=1}^{n}Dec(sk_{k},\bar{y_{k}})=\sum_{k=1}^{n}c*S_{ik}=c*S_{i}$$
3. For $c+S_{i}$:
  (16) $$\sum_{k=1}^{n}Dec(sk_{k},\bar{y_{k}})=\left(c+S_{i1}\right)+\sum_{k=2}^{n}\left(0+S_{ik}\right)=c+S_{i}$$

Moreover, the commitments of outputs can be calculated in the same way to provide the verifiability:

(17) $$Comm_{y_{k}}\leftarrow F(Comm_{1k},Comm_{2k},\ldots ,Comm_{mk},c_{1},c_{2},\ldots ,c_{m}),\;k=1,2,\ldots ,n$$

(18) $$Comm_{y}\leftarrow F(Comm_{1},Comm_{2},\ldots ,Comm_{m},c_{1},c_{2},\ldots ,c_{m})$$

The result holds since the Pedersen algorithm is additively homomorphic.

Note that we do not need open commitment after every addition gate, resulting from the fact that the addition gate can be automatically operated by *PTP* without interactions (except the final one).

(3) **Protocol Multiplication**

A multiplication gate is defined as follows:

(19) $$\begin{array}{c} \begin{array}{cc} \left\{\bar{y_{1}},\bar{y_{2}},\ldots ,\bar{y_{n}}\right\} & \leftarrow \tilde{F}\left(\bar{<S_{1}>},\bar{<S_{2}>},\ldots ,\bar{<S_{m}>}\right)\\ \bar{<S_{k}>} & \leftarrow (\bar{S_{k1}},\bar{S_{k2}},\ldots ,\bar{S_{kn}}) \end{array} \end{array}$$

The input is the encrypted shares of secret inputs $S_{i}$ (i=1,2,…,m), and there are n output values for *n* participants. The *i*-th output value $\bar{y_{i}}$ is a ciphertext encrypted by $P_{i}$’s public key $pk_{i}$. We define the multiplication gate as the computation of the multiplicative monomial:

(20) $$F\left(\cdot \right)\leftarrow \prod_{i=1}^{m}S_{i}$$

Multiplication consumes <a,b,c,d> quadruples, which is similar to Beaver triples [17]. The basic process is as follows:

(21) $$\begin{array}{c} \begin{array}{cc} x*y & =(x-a+a)*(y-b+b)\\ \; & =(x-a)(y-b)+a(y-b)+b(x-a)+ab\\ \; & =t*s*d+a*s+b*t+c \end{array} \end{array}$$

*a*, *b* and *c* are random finite field elements unknown to everyone, and *d* equals 1. *d* is necessary because it makes it possible to perform Beaver Multiplication [17] in encrypted form.

To compute $S_{i}*S_{j}$, there are three steps:

1. Obtain the encrypted shares of $S_{i}-a$ and $S_{j}-b$:
  (22) $$\begin{array}{c} \begin{array}{cc} \bar{T_{siak}} & \leftarrow \bar{S_{ik}}⊖\bar{a_{k}},k=1,2,\ldots ,n\\ \bar{T_{sjbk}} & \leftarrow \bar{S_{jk}}⊖\bar{b_{k}},k=1,2,\ldots ,n \end{array} \end{array}$$
2. Obtain *t*, *s* and ts:
  (23) $$\begin{array}{c} \begin{array}{cc} t\leftarrow \sum_{k=1}^{n} & Dec(sk_{k},\bar{T_{siak}})\\ s\leftarrow \sum_{k=1}^{n} & Dec(sk_{k},\bar{T_{sjbk}})\\ ts & \leftarrow t*s \end{array} \end{array}$$
3. Obtain the encrypted shares of $S_{i}*S_{j}$:
  (24) $$\bar{y_{k}}=\bar{T_{sijk}}\leftarrow \left(ts⊗\bar{d_{k}}\right)⊕(s⊗\bar{a_{k}})⊕\left(t⊗\bar{b_{k}}\right)⊕\left(\bar{c_{k}}\right),k=1,2,\ldots ,n$$

Now we have:

(25) $$\begin{array}{cc} \sum_{k=1}^{n}Dec(sk_{k},\bar{y_{k}}) & =\sum_{k=1}^{n}\left(ts*d_{k}+s*a_{k}+t*b_{k}+c_{k}\right)\\ \; & =ts*\sum_{k=1}^{n}d_{k}+s*\sum_{k=1}^{n}a_{k}+t*\sum_{k=1}^{n}b_{k}+\sum_{k=1}^{n}c_{k}\\ \; & =ts+s*a+t*b+c\\ \; & =\left(S_{i}-a\right)*\left(S_{j}-b\right)+a*\left(S_{j}-b\right)+b*\left(S_{i}-a\right)+a*b\\ \; & =\left(S_{i}-a+a\right)*\left(S_{j}-b+b\right)\\ \; & =S_{i}*S_{j} \end{array}$$

Therefore, the result is correct.

**Figure 2:**
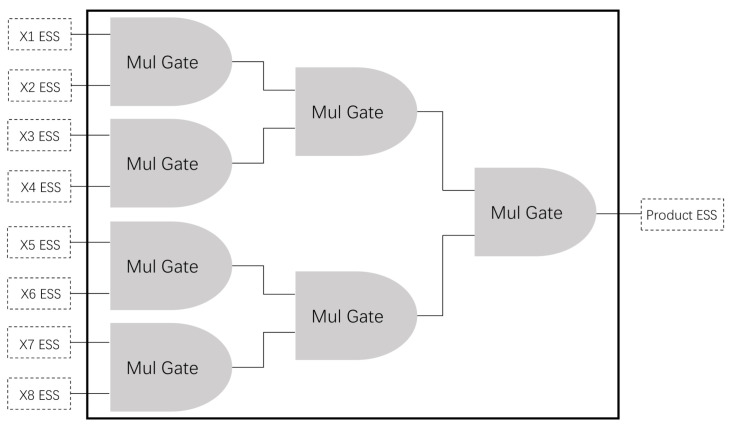
Hierarchical multiplication gate. ESS stands for “encrypted secret shares”.

To reduce the communication complexity and improve the computation efficiency, we build our multiplication gate in a hierarchical way, as shown in Figure 2. Assuming the length of a multiplicative monomial is *m*, then the depth of the multiplication gate is log(m), and computations in the same layer can be performed in parallel. The maximum depth of an arithmetic computation equals the maximum depth of monomials—log(m).

(26) $$\log\left(M\right)\leftarrow \max(\log\left(m_{1}\right),\log\left(m_{2}\right),\ldots )$$

As with the addition gate, we can perform the same computation on the blinding factor $x_{ij}$ to get the commitments of the outputs, and verify the correctness by opening commitments. In contrast, some decryption operations exist in the multiplication gate, which are performed by participants (decryptors) rather than *PTP*, leading to untrusted interactions. However, we do not need to open commitments after every multiplication gate. We can open commitments forwardly only when it fails to open the final commitments.

Any tamper behavior during the decryption phase would never succeed because *PTP* detects the attack by checking the commitments.

(4) **Protocol Output**

The output is *n* ciphertexts, *i*-th of which is encrypted by $P_{i}$’s public key $pk_{i}$.

(27) $$\begin{array}{c} \begin{array}{cc} \left\{\bar{y_{1}},\bar{y_{2}},\ldots ,\bar{y_{n}}\right\} & \leftarrow \tilde{F}\left(\bar{S1},\bar{S2},\ldots ,\bar{Sn}\right)\\ \sum y_{i} & =F(s_{1},s_{2},\ldots ,s_{n}) \end{array} \end{array}$$

The input values are secretly shared by the input gate and the computations of the addition gate and multiplication gate are based on encrypted shares, which reveal no information about input values. According to the analysis above, the corresponding commitments of the output values can be opened and checked, ensuring the result is corrected.

Participants require all the decrypted shares to recover the final result:

(28) $$Y\leftarrow \sum_{i=1}^{n}Dec(sk_{i},\bar{y_{i}})$$

#### 3.3.2. Off-Chain Preprocessing Protocol

The off-chain preprocessing phase generates multiplicative <a,b,c,d> quadruples, which is independent of inputs and can be generated in large amounts in advance.

(29) c=a∗b,d=1

*a*, *b* and *c* are random finite field elements unknown to everyone. Our off-chain protocol is similar to that in SPDZ [18], but there are some modifications to support our on-chain protocol. Like the generation of Beaver triples in [18,35], generating the quadruples is an expensive process based on somewhat homomorphic encryption (SHE), but it is independent of the onchain protocol so it can be preprocessed.

Participants distributedly generate a quadruple <a,b,c,d> where *a*, *b* and *c* are unknown to everyone and *d* equals 1. $P_{i}$ holds $a_{i},b_{i},c_{i},d_{i}$, which is the *i*-th share of <a,b,c,d>. The details of the generation are provided below.

1. Each participant $P_{i}$ generates $a_{i},b_{i}\in F_{p}^{k}$. Let
  (30) $$a:=\sum_{i=1}^{n}a_{i},\;b:=\sum_{i=1}^{n}b_{i}$$
2. Each participant $P_{i}$ computes and broadcasts
  (31) $$\bar{a_{i}}\leftarrow Enc\left(pk,a_{i}\right),\;\bar{b_{i}}\leftarrow Enc\left(pk,b_{i}\right)$$
3. All participants set
  (32) $$\bar{a}\leftarrow \bar{a_{1}}⊕\ldots ⊕\bar{a_{n}}\;,\;\bar{b}\leftarrow \bar{b_{1}}⊕\ldots ⊕\bar{b_{n}}\;,\;\bar{d}=Enc\left(pk,1\right)$$
4. All participants compute $\bar{c}\leftarrow \bar{a}⊗\bar{b}$
5. Participants set
  (33) $$\begin{array}{c} \begin{array}{cc} \left(c_{1},\ldots ,c_{n},\bar{c^{{}^{\prime }}}\right) & \leftarrow Reshare(\bar{c},NewCiphertext)\\ \left(d_{1},\ldots ,d_{n},\bar{d^{{}^{\prime }}}\right) & \leftarrow Reshare(\bar{d},NewCiphertext) \end{array} \end{array}$$
6. Participants invoke “Sacrifice” to check that indeed a∗b=c:
  (34) {true,false}←Sacrificing(<a,b,c>)

“Reshare” and “Sacrifice” are the same as that in SPDZ, and an overview of them is provided below:

**Protocol Reshare($\bar{m}$, NewCiphertext)**: Takes a ciphertext $\bar{m}\in \tilde{C}$ as the input, and outputs a new secret sharing $m_{1},m_{2},\ldots ,m_{n}\;\in \tilde{M}$ and a new “fresh” ciphertext $\bar{m^{{}^{\prime }}}\in \tilde{C}$ such that $Dec(sk,\bar{m^{{}^{\prime }}})=\sum_{i}m_{i}$.

**Protocol Sacrifice (<a,b,c>)**: Takes a triple and outputs whether the triple satisfies c=a∗b.

### 3.4. On-Chain Secure MPC in Hyperledger Fabric

#### 3.4.1. A Brief Introduction to Hyperledger Fabric

In Hyperledger Fabric, peers have access to the ledger and execute specific programs—chaincode (smart contract). Clients send transaction proposals to one or more peers with setting the endorsement policy. The endorsing peers then execute the chaincode to decide whether the transaction should be endorsed. If so, endorsing peers change the state on the ledger according to the targeted chaincode. An endorsement policy is a condition on what endorses a transaction [15]. Thus a pre-specified endorsement policy is necessary when installing specific chaincode. Some example endorsement policies include “at least one from among the four organizations” and “all organizations”.

A critical detail is that all endorsing peers see identical transaction proposals (no matter whether they would be accepted in the next phase). Transactions are executed and verified in the endorsement phase so we run our on-chain compute protocol during that phase.

#### 3.4.2. A Crucial Additional Component

A participant is a data provider who connects to the blockchain network by a client. Any participant can own at least one peer, through which he can join in one or more organizations in Fabric. As mentioned earlier, participants store encrypted inputs in the ledger (*PTP*). As inputs are encrypted by different public keys, we need to deal with the question of how to perform decryption while executing multiplication gate. Unlike the solution of Benhamouda [31], we do not require “privileged clients” or a “helper server”. Participants each store their private keys locally in one of their peers that serves as a “decryptor”, which is responsible for processing decryption requests during the endorsement phase. A decryptor does not need to be an endorsing node, but it must remain online during the endorsement phase.

The decryptor utilizes cache technology to avoid processing duplicate decryption requests since different endorsing peers execute identical transactions during the endorsement phase. When receiving same requests, decryptors immediately obtain the result from the cache and respond. A decryptor is a pluggable component, so we do not need to do many code modifications to Fabric.

Moreover, a decryptor helps to eliminate communication interactions among participants and the blockchain. For as long as the decryptors are online, all a participant needs to do is encrypt and submit his input and wait for the outputs.

#### 3.4.3. Implementation Details

The details of our secure MPC protocol are explained in Section 3.3. Here, we describe how the protocol can be integrated into Fabric.

Our MPC library was written in C++, and we used Java chaincode in Fabric. To call the MPC library in Java chaincode, we used JNI [36] technology, which allows us to call C++ code from Java. We used a customized Docker container, fabric-javaenv, where our MPC library was installed.

In regard to the implementation, there are two problems. The first is how the chaincode find the correct decryptors that it needs to communicate with. The second problem is how to tell decryptors the progress of the computation so it can verify whether the decryption request is valid and whether to accept the request. To solve the first problem, we opted for simple solutions where decryptors’ addresses (IP address and port) are dynamically written into the ledger as transaction proposals by clients in advance. The solution requires no code modifications to Hyperledger Fabric.

To solve the second problem, we need some definitions to the data packet of the decryption request:

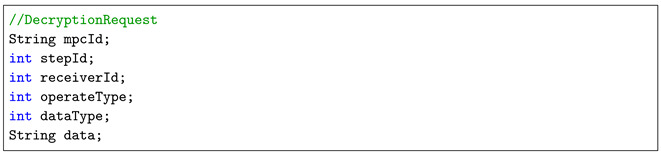


The *mpcId* is a unique id used to distinguish different computation requests. The *stepId*, which increases from 0, represents the progress of the current computation and is controlled by the chaincode. With *mpcId* and *stepId*, decryptors are able to check whether the request corresponds to a correct step by calculating the commitments locally and opening commitments after decrypting the data. If succeeding in opening commitments, decryptors store them into the cache and respond. If it fails, they ignore the request. Note that the results of decryption are some random values, which reveal no information about input values.

## 4. Security Analysis

In this section, we discuss the security issue of our solution. As we pointed out at the beginning, the most critical problem is the leakage of the input data. No one should be able to learn anything except for their own inputs and outputs.

**Data privacy and Privacy control**: The combination of homomorphic encryption and secret sharing allows computations to be performed on the encrypted shares. $P_{i}$ encrypts different share using different participants’ public key so that other participants can only see their own authorized share, which are meaninglessly random values and would never reveal any valid information about $P_{i}$’s input value $S_{i}$. No secret can be reconstructed without decrypting all the shares. What’s more, intermediate data (*t* and *s*) generated by decryptors are meaninglessly random values if no participant knows *a* or *b* of the <a,b,c,d> quadruple (actually it is). According to our settings, $P_{i}$ holds the *i*-th share of *a* and *b*, and no one knows *a* or *b*. Commitment Comm(x,a) is theoretically private since there exist many possible combinations of *x* and *a* that would generate the same Comm. Even with the same private data *a*, Comm(x,a) would be totally different when different values of *x* exist. If *x* is truly random, an attacker would be completely unable to figure out *a* [37].

**Resistance to collusion attacks**: In our solution, a secret is broken into shares by the additive secret sharing scheme and then different shares are encrypted by different participants’ public key. The threshold of the additive secret sharing scheme is *n*, which means our scheme remains safe even there are up to n−1 conspiracy adversaries among the participants. *PTP* automatically verifies the data decrypted by decryptors by opening commitments so that decryptors fail upon tampering.

**Publicly verifiable MPC**: Participants can verify whether the input value is valid and whether the dealer is honest by opening commitments. Due to commitments being public and automatically computed by *PTP*, everyone can check the correctness of the result by opening commitments. Although there are interactions in the multiplication gate, an active attack can be defended against, since any tamper behavior during the decryption phase would never succeed because *PTP* detects the attack by verifying the commitments.

**Trust model**: The trust model of the proposed on-chain secure MPC protocol depends on the endorsement policy set when installing the chaincode. For example, if the trust model allows no more than *k* adversarial participants, we can simply set an endorsement policy that demands at least k+1 endorsing peers, guaranteeing that transactions tampered by some adversaries will never be successfully endorsed.

## 5. Experimental Results and Discussions

Our running environment was ubuntu 18.04, Intel(R) Core(TM) i5-6400 CPU @ 2.70 GHz, 16 GB of RAM and Hyperledger Fabric v1.4.0. We used the customized Docker container fabric-javaenv, as mentioned in Section 3.4.3, for chaincode execution. Peers and chaincode were all running on separate Docker containers on the same machine. We communicated with the blockchain network using Hyperledger Fabric SDK for Java v1.4.0. In all of our experiments, each organization had only a single peer and peers belonged to different participants. We set “all organizations” as the endorsement policy, meaning that the number of participants equals the number of computing nodes.

### 5.1. Comparison of Running Time Based on Different Key Sizes

In this experiment, we studied the impact of key size in Paillier Cryptosystem on running time. We jointly computed the weighted average of inputs from 10 participants and set i as the weight of $P_{i}$. Thus, the function is

(35) $$F\left(\cdot \right)=\frac{\sum_{i=1}^{n}i*S_{i}}{\sum_{i=1}^{n}i},n=10$$

**Figure 3:**
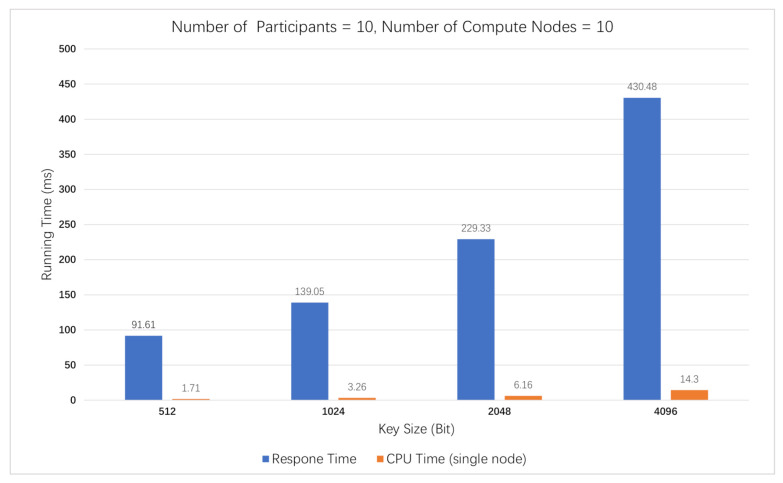
Response time and CPU time based on different key sizes. The number of computing nodes was ten.

Figure 3 shows the total response time for securely computing the above function based on our secure MPC protocol, and the CPU time in a single node (ignoring the consensus time or communication time).

(1) It can be seen that the total response time ranges from 91.61 milliseconds for 512 bits to 430.48 milliseconds for 4096 bits, and the CPU time in a single node ranges from 1.71 milliseconds for 512 bits to 14.3 milliseconds for 4096 bits. Both response time and CPU time grow with the increase in key size. This is normal because of the larger ciphertext and the increasing computational complexity, but it leads to higher security.

(2) In addition, the CPU time is negligible compared to the total response time. Even when the key size is 4096 bits, the CPU time accounts for less than 4% of the total response time, and most of the time is spent on communication and reaching consensus, meaning that the performance has a lot of room to grow with the increasing optimization of communication channels.

The NIST [38] recommends 2048-bit key as the standard key size. Therefore, for simplicity, we use a 2048-bit key in the following experiments.

### 5.2. Comparison of Response Time Based on Different Schemes

**Figure 4:**
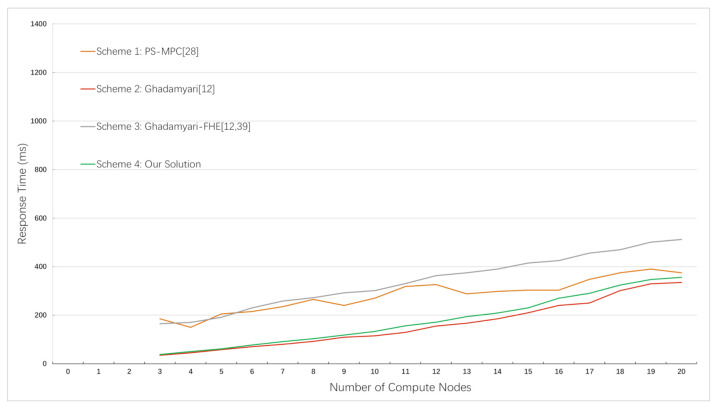
Response time based on different schemes and different numbers of computing nodes.

In this experiment, we compared the response times for securely computing the sum of two inputs with an increase in the number of participants (computing nodes) based on three other schemes and our solution in this paper. The results are shown in Figure 4.

Scheme 1 offloads private and intensive computations to an off-chain network. Similar to our solution, scheme 2 runs the secure MPC protocol on-chain and uses the Paillier cryptosystem. Scheme 3 has the same architecture as that in scheme 2, but we replace its homomorphic encryption scheme with BFV [39] to support multiplicative homomorphic operations by using the SEAL [40] (an open-source library contributed by Microsoft).

(1) As illustrated, when there are no more than 20 computing nodes, our solution takes less time compared to scheme 1, but it seems that scheme 1 has a better performance when the network is large. This is because scheme 1 only stores MACs [18] and commitments on the blockchain for verification. Additionally, they separate the verification from computation, which is offloaded to an off-chain network. The architecture makes it efficient to perform computations in a large network, but it has similar performance in a small one. Differently, we take the on-chain way. When peers finish endorsement, both computations and verifications are done. The one-step method saves the performance. When the network grows, the overhead time required for consensus increases gradually, resulting in long latency. Therefore, our solution is more suitable for computations in permissioned blockchain, such as Hyperledger Fabric, which meets the initial goal in this paper.

(2) We can see that scheme 2 consumes the least time. However, compared with our solution, the time consumption difference is quite small. Both of us use the Paillier cryptosystem, but organizations in scheme 2 encrypt values using the same key. Although they use access control list (ACL) rules to control access to the ledger, there still exists a conspiracy between the key owner and smart contract invoker. Differently, our scheme remains safe even there are up to n−1 conspiracy adversaries according to the above analysis. What is more, scheme 2 only supports addition operations or scalar multiplication operations, but we are able to perform multiplication on ciphertext, which is deserved at the expense of a small loss in the computational performance.

(3) Figure 4 shows that scheme 3 consumes the most time among all solutions based on blockchain, because the performance of fully homomorphic encryption (FHE) is currently quite inefficient. Many experts and scholars are making efforts to find a balance between utility, protection and performance.

(4) In addition, we can see that, even for 20 nodes, the running time of our solution is about 350 milliseconds, which is longer than the time it takes to commit a block (concretely 2254 milliseconds for ten organizations in our experimental environment).

## 6. Conclusions and Future Work

In this paper, we proposed a publicly verifiable, secure MPC protocol consisting of two parts: an on-chain computation phase and an off-chain preprocessing phase. The scheme has the following advantages: (1) Privacy confidentiality: all input values and output values are in encrypted form and everyone learns nothing except his own input secret and output values. (2) Correctness and verifiability: Computations can be publicly executed and are controlled by no one. All the steps during computation can be publicly verified. We also described how the proposed secure MPC protocol can be integrated into Hyperledger Fabric, which helps to handle transactions that rely on confidential data owned by different participants. The scheme greatly guarantees the privacy of smart contract execution. The experiments showed that our solution had good execution efficiency. Meanwhile, most of the time taken to complete the protocol was spent on communication so the performance has a great deal of room to grow.

The expensive communication costs limit our solution’s scalability to a larger network. In the future, we will explore the ways in which communication times and data exchange during computation can be reduced, or try to build a more efficient P2P communication channel for the decryptor. Moreover, using the proposed method to solve practical problems such as statistical analysis on credit data with guaranteed privacy is also among our plans.

## Abbreviations

   The following abbreviations are used in this paper:

MPC	Multi-party Computation
PTP	Publicly Trusted Processor
